# Understanding translational research in schizophrenia: A novel insight into animal models

**DOI:** 10.1007/s11033-023-08241-7

**Published:** 2023-01-24

**Authors:** Jonaid Ahmad Malik, Zahid Yaseen, Lahari Thotapalli, Sakeel Ahmed, Mohd Farooq Shaikh, Sirajudheen Anwar

**Affiliations:** 1 Department of Pharmacology and Toxicology, National Institute of Pharmaceutical Education and Research, Guwahati, India; 2 Department of Biomedical Engineering, Indian Institute of Technology Ropar, Rupnagar, India; 3 Department of Pharmaceutical Biotechnology, Delhi Pharmaceutical Sciences and Research University, Delhi, India; 4 Department of Pharmaceutical Sciences, JNTU University, Anantapur, India; 5 Department of Pharmacology and Toxicology, National Institute of Pharmaceutical Education and Research, Ahmedabad, Gujarat 382355 India; 6 Neuropharmacology Research Strength, Jeffrey Cheah School of Medicine and Health Sciences, Monash University Malaysia, 47500 Bandar Sunway, Selangor Malaysia; 7 School of Dentistry and Medical Sciences, Charles Sturt University, Orange, 2800 New South Wales Australia; 8 Department of Pharmacology, College of Pharmacy, University of Hail, Hail, 81422 Saudi Arabia

**Keywords:** Animal models, Schizophrenia, Animal models of schizophrenia, Schizophrenia models, Psychotic diseases

## Abstract

Schizophrenia affects millions of people worldwide and is a major challenge for the scientific community. Like most psychotic diseases, it is also considered a complicated mental disorder caused by an imbalance in neurotransmitters. Due to the complexity of neuropathology, it is always a complicated disorder. The lack of proper understanding of the pathophysiology makes the disorder unmanageable in clinical settings. However, due to recent advances in animal models, we hope we can have better therapeutic approaches with more success in clinical settings. Dopamine, glutamate, GABA, and serotonin are the neurotransmitters involved in the pathophysiology of schizophrenia. Various animal models have been put forward based on these neurotransmitters, including pharmacological, neurodevelopmental, and genetic models. Polymorphism of genes such as dysbindin, DICS1, and NRG1 has also been reported in schizophrenia. Hypothesis based on dopamine, glutamate, and serotonin are considered successful models of schizophrenia on which drug therapies have been designed to date. New targets like the orexin system, muscarinic and nicotinic receptors, and cannabinoid receptors have been approached to alleviate the negative and cognitive symptoms. The non-pharmacological models like the post-weaning social isolation model (maternal deprivation), the isolation rearing model etc. have been also developed to mimic the symptoms of schizophrenia and to create and test new approaches of drug therapy which is a breakthrough at present in psychiatric disorders. Different behavioral tests have been evaluated in these specific models. This review will highlight the currently available animal models and behavioral tests in psychic disorders concerning schizophrenia.

## Introduction

Schizophrenia is considered a complicated, chronic psychological disorder characterized by a batch of symptoms, including hallucinations, delusions, disordered speech or behavior, blunted affect, anhedonia, alogia, apathy, and other defective cognitive abilities. The acute relapses of this disease result from the aggravation of positive psychotic symptoms, while the chronic disabilities result from cognitive and negative symptoms (apathy, social withdrawal etc.) [1].

### Neuropathology and associated molecular pathways

Anomalies associated with the transmission of neurons have been considered the basis for understanding the pathophysiology of schizophrenia. Most of these theories focus on the imbalance of neurotransmitters including dopamine (DA), glutamate (Glu), and serotonin (5-HT). Other theories also focus on glycine, GABA, and aspartate as part of the neurotransmitter imbalance in schizophrenia. In 1960’s, the discovery of the antipsychotic actions of chlorpromazine (DA blocker) led to the understanding of pathology associated with schizophrenia. More development was made to improve antipsychotic drugs primarily guided by the fact that schizophrenia is partially associated with hyperdopaminergic state [2].

#### Dopamine hypothesis

**Fig. 1:**
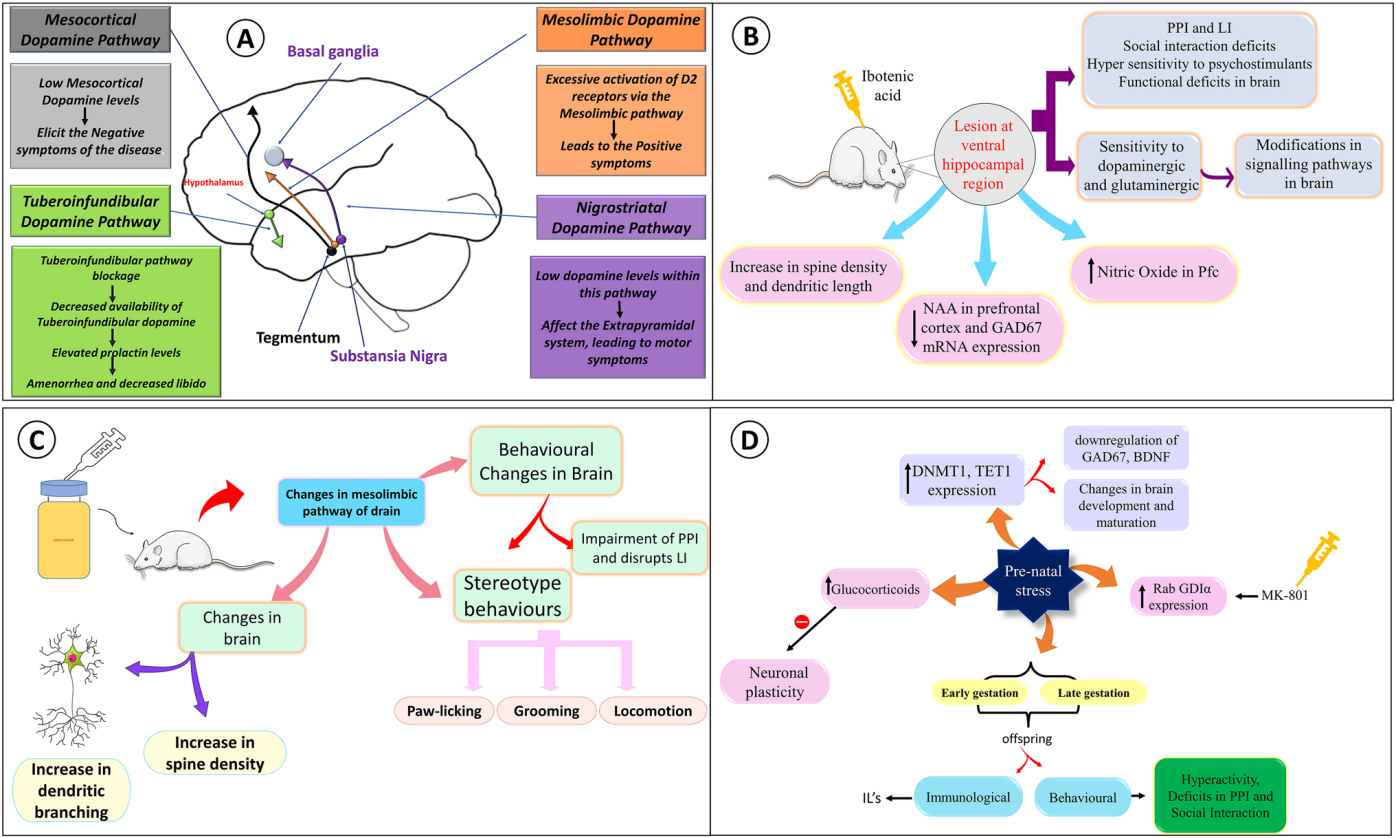
**A** Pathophysiology of schizophrenia according to dopamine hypothesis, **B** Depicts amphetamine associated morphological and behavioral changes in mesolimbic pathway of brain, **C** Depicts behavioral changes and pathways associated with NVHL, **D** depicts prenatal stress model

Many of the symptoms of schizophrenia is thought to be connected with the strange behavior at DA receptor sites (specifically D2). To understand this, four pathways as depicted in Fig. 1A are necessary to understand the phenomenon. Hyperactivity of DA in the Mesolimbic pathway originating from ventral tegmental area to limbic areas is considered to play a role in the positive symptoms of schizophrenia. Low DA level in mesocortical pathway originating from ventral tegmental area to cortex is associated with cognitive deficits and negative symptoms. Low levels of DA in nigrostriatal pathway originating from substantia nigra and ends in the caudate nucleus, compromises the extrapyramidal system which contributes to motor symptoms. Obstruction or decrease of DA level in the tuberoinfundibular pathway (originates from hypothalamus to pituitary gland) contributes to increased prolactin levels and results in galactorrhea, amenorrhea, and reduced libido [1]. Current findings have shown contrasting results as DA hypothesis seems insufficient in explaining symptoms (excluding positive symptoms), due to involvement of other receptors (D1 and D3) and neurotransmitters such as serotonin and glutamate playing role in alleviating the cognition and negative symptoms. The negative symptoms such as apathy, anhedonia, and alogia seems the result of decreased D1 receptor activation in the prefrontal cortex and hypoactivity of the nucleus caudatus. Dysfunction in D (3)-receptors might also be linked with the negative symptoms of schizophrenia [3].

#### Glutamate hypothesis

In schizophrenia patients, early finding of low levels of glutamate in cerebrospinal fluid originates glutamate hypothesis. Observing the effect of NMDA antagonist drugs (like ketamine and phencyclidine) imitates many features (positive, negative and cognitive symptoms) of schizophrenia. This hypothesis postulates that the primary deficiency of NMDA glutamate receptor causes this disease's positive, negative, and cognitive symptoms1.Many roles are played by Glu as neurotransmitter including attention, cognition, perception, synaptic pruning, axonal guidance and plasticity (in utero and during adolescence). Many studies concur with the glutamate hypothesis including postmortem studies showing changes in NMDA receptors and related proteins, and recent PET imaging (suggests reduced hippocampal NMDA receptor binding) [4]. The possible mechanism was theoretically credited to hypoactive glutamate NMDA receptors (present on GABA interneurons in the PFC) instigating positive symptoms (delusions and hallucinations) through increased downstream dopaminergic mesolimbic activity while as producing negative symptoms (cognition impairment) by decreased downstream dopaminergic mesocortical activity. This hypoactive glutamate NMDA receptors are further attributed to abnormal development of neurons present in prefrontal cortex (PFC) [5].

#### Serotonin hypothesis

This hypothesis emerged as a result of the discovery by Swiss chemist Albert Hoffman, that lysergic acid diethylamide seemed to intensify the effects of serotonin in the brain. The pathophysiology has been related with chronic prevalent stress-induced serotonergic hyperactivity in the cerebral cortex of schizophrenic patients, especially in the anterior cingulate cortex (ACC) and dorsolateral frontal lobe effecting glutamate signalling causing neuronal hypometabolism, synaptic atrophy and grey matter loss. Thus, normal DA supply through impaired ACC causes positive symptoms while as frontal lobe hibernation causes negative symptoms and cognitive dysregulations. This hypothesis of serotonergic hyperactivity is supported by NMR spectroscopy, PET data with serotonergic ligands, peripheral exhaustion of phospholipids, and serotonergic 5-HT_2A_ receptors being linked to phospholipase A2 [6]. Further research contributed to development of medications like risperidone and clozapine, that appeared to work by dopamine (D2) and serotonin (5-HT2A) receptor blockage, thus reducing the course of the disease. These drugs were in contrast to other older and typical medications, that typically used to block dopaminergic receptors. This serotonin blocking action of atypical antipsychotic drugs seemed very effective in alleviating both positive and negative symptoms of schizophrenia [1].

Compared to the DA hypothesis, the serotonin hypothesis is still considered less convincing in considering the pathophysiology of schizophrenia. As there has been interrelations found between negative symptoms and 5-HT receptors, this hypothesis was given to correlate the involvement of 5-HT receptors in schizophrenia [7]. All three pathways have been considered interlinked with one another as all are involved in inducing hallucinations and delusions (core symptoms of schizophrenia).

Other than above theories, many different approaches have been taken to understand profoundly the pathophysiology of schizophrenia including glial cell contribution, cannabinoid hypothesis, etc.

#### Glial cell hypothesis

The contribution of glial cell dysregulation to schizophrenia is doubtful, but research findings cannot be ruled out. The gliocentric model of schizophrenia suggests all four types of glial cells are involved in the pathology of schizophrenia. This hypothesis focuses on inappropriate timing of maturation of microglia during fetal development, negatively effecting the differentiation of glial progenitor cells resulting in deficit astrocytes and oligodendrocytes in terms of number, morphology, and functionality. Reduced synaptic coverage and abnormal buffering of glutamate and potassium are repercussions of astrocyte differentiation failure while as immature oligodendrocytes cause hypomyelination and loss of white matter integrity. Glial cell hypothesis further suggests that primary event could be failure of glial differentiation followed by astrogliosis and microgliosis as secondary events resulting due to disturbances in network connectivity, changes in neuromodulator release, homeostatic failure of glutamate and potassium regulation, neuroinflammation, drug related effects and sleep deficits [8]. More research is required to link the glial cell contribution with schizophrenia and in future, it may provide some potential novel insights to understand pathology of schizophrenia and at the same time can uncover the new drug therapy approaches.

#### Cannabinoid hypothesis

Cannabinoid hypothesis suggest that the alterations in the endocannabinoid system may contribute to pathogenesis of schizophrenia. Resemblances between cannabis-induced neurophysiological abnormalities and those witnessed in schizophrenia led to cannabinoid hypothesis. Cannabinoids has the potential of inducing the range of symptoms such as transient subjective, behavioral, cognitive, and psychophysiological effects known to be associated with schizophrenia. These effects has been attributed to decreased P300 amplitude resulting into acutely disrupting cortical processes responsible for context updating and the spontaneous orientation of attention [9]. Other study reported aberrations with endocannabinoid and endovanilloid signaling through mesocorticolimbic pathways may promote schizophrenia like symptoms. Exploring through these novel pathways would likely give therapeutic privileges in future for treatment of psychotic conditions like schizophrenia [10].

### Role of dopamine, glutamate, serotonin, and γ-aminobutyric acid (GABA) in schizophrenia

Neurotransmitters involved in schizophrenia are: Dopamine, glutamate, GABA and serotonin. Dopamine plays an important role in schizophrenia. According to the dopamine hypothesis, the increase or decrease in dopamine levels occur in the mesolimbic area and PFC respectively, which inflicts positive and negative symptoms. Hallucinations and delusions come under positive whereas lack of motivation and anxiety come under negative symptoms. As, both the symptoms has been linked to DA imbalance, but few studies suggest that only positive but not negative symptoms can be related to DA hypothesis due to lack of any firm explanation [11]. Post mortem studies of dopamine showed decreased cortical dopamine innervation and alterations in D3 receptor splicing and increased D4 receptor like binding [12].

Glutamate is an amino acid and excitatory neurotransmitter in brain. There are two types of Glu receptors: metabotropic and ionotropic receptors. These ionotropic receptors are divided into three types i.e. N-methyl-D-aspartate (NMDA), α-amino-3-hydroxy-5-methyl-4-isoxazolepropionic acid (AMPA) and kinase receptors, among these NMDA receptors are associated with schizophrenia [13]. When there is dysfunction or hypoactivity of NMDA receptors, there will be an increase in glutamate levels, leading to schizophrenia. From post mortem studies, it could be concluded that decreased hippocampus (HC) AMPA and kinase receptor expression and altered glutamate fibers in cingulate cortex can cause schizophrenia [12]. Metabotropic receptors modulate the NMDA receptors which leads to schizophrenia. Genes associated with the glutamatergic system such as dysbindin, neuregulin, d-amino oxidase (DAAO), G72/G30, glutamate transporters can also lead to schizophrenia on their activity. The polymorphism can also cause schizophrenia in metabotropic receptor gene (mGluR7) [14]. Dysfunction in Glu levels can cause negative symptoms such as behavioral and cognitive defects [13].

GABA is an inhibitory neurotransmitter and dysregulation of NMDA receptors affects the Glu and GABA pathways i.e. decreases the GABA levels [11]. Due to decrease in GABA levels, causing increase in dopamine levels which further causes schizophrenia [15]. Symptoms such as disturbances in emotional, behavioral and cognition are caused when GABA levels are decreased in frontal cortex of brain. In post mortem studies, the findings include increased GABA_A_ receptor binding in limbic areas, altered expression in frontal cortex GABA_A_ receptor subunits, altered density of cingulate GABAergic cells, decreased expression of glutamic acid decarboxylase [12] and decreased GAD67 mRNA in nucleus accumbens (NAcc) and orbital frontal cortex regions of brain [16].

5-HT is widely distributed in brain as receptors associated with it are of 14 different subtypes. Among these subtypes 5HT_1A_, present in cortical and HC. 5HT_2A,_ present in peripheral tissues, 5HT_4_ and 5HT_6_ are also responsible for cognitive functions [15]. Decrease in the 5HT_2A_ and increase in 5HT_1A_ receptor density causes schizophrenia. Schizophrenia can also be caused due to polymorphism of 5HT_2A_ receptor gene [13]. Post mortem findings show decreased frontal cortex 5-HT_2A_ receptor expression and increased 5-HT transporter affinity [12].

### Molecular targets for schizophrenia

With the discovery of phenothiazines (chlorpromazine) in 1950’s, new era of antipsychotics to treat psychotic disorders began. Serendipitous discovery of butyrophenones as antipsychotic by Paul Janssen led to the birth of a new class of antipsychotics, including haloperidol, etc. Until 1958, other drugs were also discovered like thioridazine and trifluoperazine came into use, but all these drugs had extrapyramidal symptoms (EPS). These drugs were called as first-generation antipsychotics (FGA). Researchers looking for new drug with lesser side effects until 1958, Swiss laboratory synthesized tricyclic compounds as anti-depressant drugs including clozapine. Later, clozapine was used to treat schizophrenia with fewer side effects than FGA. Clozapine was considered the earliest second-generation antipsychotic (SGA) or atypical antipsychotic drug followed by the arrival of other atypical drugs, including olanzapine, risperidone, quetiapine, ziprasidone, and other similar drugs [17]. As the mechanism of action of early antipsychotic (FGA) drugs were mostly based on antagonistic effect on DA receptor (specifically D2) and low 5-HT antagonism (specifically 5-HT2A) while as atypical antipsychotic (SGA) drugs shows high antagonistic effect on 5-HT (5-HT2A) receptors and variable (low to moderate to high) antagonistic effect on D2 receptor [1]. Novel drugs targeting different sites like muscarinic, nicotinic, cannabinoids, glutamate etc. which are currently in clinical and preclinical trials are shown briefly in Table 1.

**Table 1:** Depicts the different schizophrenia targets and Novel drugs that targeted the same and are in clinical trials

Name of target	Target involved	Effect	Drugs in clinical trials	Clinical status	References
Dopamine	D2	Modulates DA signaling downstream of receptor activation in striatum pathway	TAK-063, a PDE10A inhibitor hydrolyzes both cyclic adenosine monophosphate and cyclic guanosine monophosphate and alleviates general symptoms	Phase 2	(NCT02477020)
Serotonin	5-HT2A and Sigma2 receptor antagonist	Antagonist	Roluperidone (also known as MIN-101) shows its efficiency in alleviating negative symptoms	Phase 3	(NCT03397134)
Glutamate	1. Metabotropic glutamate receptors or mGlu A. Group I mGlu (5) Group II mGlu receptors (mGlu(2) and mGlu(3))	Agonists	Selective positive allosteric modulators (PAMs) on mGlu1 receptor shows promising results in improving positive symptoms mGlu5 receptor PAMs acts by alleviating neuroinflammation, Other subtypes such as mGlu2 receptor PAMs, orthosteric mGlu2/3 receptor agonists and mGlu4 are showing anti-psychotic effects in animal models	Preclinical	[18]
	2. Ionotropic glutamate receptor or iGluR	Positive allosteric modulator	BIIB104, currently in phase 2 targeting cognitive impairment associated with schizophrenia	Phase 2	(NCT03745820)
	3. Glycine site of the NMDAR	Antagonist	D-cycloserine has been shown some efficacy in treating negative symptoms of schizophrenia as well as Memantine for cognition and psychopathology	Phase 3	(NCT00148616), (NCT00000371)
Acetylcholine receptors	Muscarinic receptor M1 and M4	Agonist and antagonist	KarXT, a combination of xanomeline (novel muscarinic agonist) and trospium (a muscarinic antagonist), is believed to reduce psychosis and improve cognition	Phase 3	(NCT04659161)
	Α7 Nicotinic receptors	Agonists	Primarily focusing on the alpha7 nicotinic agonism (3–2,4 dimethoxybenzylidene anabaseine (DMXB-A)) as a strategy to treat cognitive symptoms in schizophrenia	Phase 2	(NCT00100165)
Cannabinoids	Dopaminergic, GABA, and glutamatergic neurotransmission	Psychotogenic	THC, principal constituent present in Cannabis may be psychotogenic (induces psychotic symptoms)	NA	(NCT04105231)
	Cannabinoid (CB) receptors	Indirect antagonist	Cannabidiol has been found to prevent psychotic-like symptoms induced by high doses of THC	Phase 2	
Orexin system	Orexin receptors	Agonist and antagonist	TCS 1102 (antagonist) and orexin A and B peptides (agonist) were given back-to-back, to see the indirect effect on dopamine system function via paraventricular nucleus of the thalamus (PVT)	Preclinical	[19]

## Behavioral tests used to evaluate the models of schizophrenia

Animals used for studying schizophrenia includes rat, mice and primates. Many behavioral models are used to evaluate the models of schizophrenia for their validity, relevance and reliability but very few have been studied in depth for parameter check. The behavioral tasks or paradigms used to evaluate the models of schizophrenia includes latent inhibition, prepulse inhibition, 5-choice serial reaction time task, working memory, object and spatial recognition memory, reversal learning and attentional set shifting task, forced swim test, sucrose preference test, social interaction test and open field test. The overview of all the parameters used to evaluate is briefly explained below.

### Latent inhibition (LI)

Latent inhibition is pre-exposing the subject to a conditioned stimulus without an unconditioned stimulus inhibits conditioning when the stimuli are subsequently paired. LI in simple terms, refers to a cognitive process in which frequent non-rewarded experience of a stimulus delays the capability of that stimulus to enter into new learnings eventually. As LI has been found diminished in schizophrenia subjects, so LI becomes one of the parameter to evaluate the models of schizophrenia. Different pharmacological modifications can create two contrast abnormalities in LI i.e., disrupted LI (model of the positive symptoms) and abnormally persistent LI (model of the negative symptoms) [20].

### Prepulse inhibition (PPI) of startle

Prepulse inhibition (PPI) of startle is a measure of sensorimotor gating that is defective in schizophrenia. PPI is a neurological process in which a low-intensity prepulse (stimulus) inhibits the startle response by a strong reflux eliciting pulse (stimulus). To understand the mechanism of the brain based inhibitory process and their deficits in neuropsychiatric disorders like schizophrenia, PPI is occasionally used. PPI deficiency is considered an endophenotype of the disease and is reminiscent of schizophrenia subjects [21]. Many PPI deficiency models are available for studying positive and negative symptoms of schizophrenia.

### 5-choice serial reaction time task (5-CSRTT)

5-choice serial reaction time task (5-CSRTT) method is employed to check the visual attentional process in the rats. It requires a subject (rat or mice) to recognize brief flashes of light coming from any one of five holes of operant chamber, by using a nose poke, followed by a reward for the same. For this test, the subjects are trained for at least 30–40 times daily sessions and eventually learn to react in the appropriate hole with a given amount of time. If the subject fails to react on time, or fails to react, or reacts in the wrong hole, no reward is delivered and a short period of darkness is awarded as punishment. In general, this behavior test measures the subject's attention, impulsivity, and vigilance [22].

### Working memory (WM)

Working memory (WM) is applied to animal cognition and is defined as a short-term memory for stimuli and spatial locations usually used within a testing session but not in between testing sessions. It measures the ability to hold information for short term to resolve complex cognitive tests related to visual, spatial, and olfactory functions. Working memory impairment is considered the core domain associated with schizophrenia and is usually connected with abnormal dorsolateral PFC activation [23].

### Object and spatial recognition memory (OSRM)

Object and spatial recognition memory (OSRM) measures recognition (cognition ability) via the animal’s integral capacity to explore new environments and objects using variety of tests like novel object recognition test, object location test etc. Schizophrenia patients are frequently associated with aberrant OSRM. Compared to normal subjects, both spatial and object recognition memory are highly correlated with each other in schizophrenia subjects. Schizophrenia also shows more changeability in spatial than object recognition memory performance. [24].

### Forced swim test (FST)

Forced swim test (FST), is a behavioral test where an animal is submitted in an inescapable transparent cylinder with water filled in it, to induce the feel of despair and hopelessness. During their stay in tank, there escape related movement is measured. Primarily used to evaluate the antidepressant efficacy of new drugs, and experimental manipulations with purpose of rendering or preventing depressive like conditions. Few recommendations support the use of test to assess antipsychotic efficacy for negative symptoms, as it has been reported that typical antipsychotic can reverse the increase in immobility time induced by blocking NMDA receptor in FST. Few reports showed less promising results that FST can be used to evaluate the modeling of negative symptoms in NMDAR antagonist animal model of schizophrenia [25].

### Sucrose preference test (SPT)

Sucrose preference test (SPT), is a reward-based test where some mice are presented to dual option of sipper tubes, one containing sucrose solution and another with drinking water. As mice are born with a strong affinity towards sweet products and continuous sucrose consumption causes anhedonia (reduced ability to experience pleasure) in our subjects which can be reversed by administering antidepressants. Sucrose preference test determines negative symptoms (anhedonia) associated with depression and schizophrenia. Anhedonia is defined as reduced ability to experience pleasure and is commonly associated with schizophrenic subjects. Sucrose preference test measures the hedonic or rewarding effect of sucrose consumption [26].

### Social interaction test (SIT)

Social interaction test (SIT), was primarily created to quantitatively assess the social behavior in mice shown during encountering the standard subject i.e. a foreign subject of the same species, shape, age and weight. Every species when placed with similar species has the craving to interact with one another, which is associated with social behavior. Behavioral actions are seen when social animals like rodents are placed in a cage. Actions including both frisky and aggressive acts like chasing, crawling over/under, boxing, wrestling, pouncing, biting, sniffing (anogenital), social grooming, etc. are seen [26]. The test duration is 10 min during which many behavioral parameters are recorded including avoidance behavior, approach behavior, aggressive behavior, attacking, locomotor activity, self-grooming etc. The measurements calculated during the test include the total time, number of events, and the percent of animals that showed specific behaviors [27]. In reference to schizophrenia, reduced social interaction is a negative symptom associated with schizophrenia subjects. Thus, this test is used for evaluating negative and cognitive symptoms in schizophrenia.

### Open field test

Open field test, measures the voluntary locomotor actions in an open field arena. Here the subjects are placed in an open field for a given time of 15 min to 2 h. Parameters which reflects locomotive function, are measured like horizontal and vertical activity, movement and rest time, and total distance travelled. The relation of this test is primarily based on the fact that many drugs like amphetamine and phencyclidine (psychostimulant drugs) efficiently induces hyper locomotor activity and that antipsychotic drugs oppose the same effect by producing sedative effects, thus reducing activity. Amphetamine and phencyclidine is used to model the positive and negative symptoms respectively [28].

## Gating deficits

Studies have shown that HC is the main mediator for sensory gating. P50 gating measures the startle response of PPI, where p stands for positive sign and 50 stands for delay of onset of stimulus in milliseconds [29]. Using auditory paired click task to measure the sensory gating, which contains P50, N100 and P200 is used to assess sensory gating deficits [30]. In this auditing a dual click task of 2 successive sounds are observed within 500 ms, the ratio of these two sounds is called P50 gating ratio [29]. All these gatings are related to filtering mechanisms i.e. P50 is related to information processing, N100 is related to triggering of attention and P200 is related to the allocation of attention [30]. P50 is used for studying neurodevelopmental and the new genetic effects during birth [31]. P300 responses are associated with cognitive processing [29], whereas N100 is associated with auditory response [32].

## Animal models of schizophrenia

Animal models monitor the molecular and structural changes that causes the diseases and also acts as preclinical tools for investigating the disease. Animal models of schizophrenia can be divided into pharmacological, and non-pharmacological (including genetic models etc.) [33]. All animals should have adequate and appropriate validity which includes face validity (symptoms homology), construct validity (pathology), predictive validity (expected pharmacological response). Face validity refers the similarity in behavior of animal model and disorder. Construct validity mimics underlying mechanisms or etiology of disease. Predictive validity refers to treatment of the diseases [34].

### Pharmacological models

Pharmacological models are the animal models to which the substances are administered in either acute or chronic doses and monitoring the changes in the neurotransmitter (receptor) systems. Mainly there are two types of pharmacological models in schizophrenia i.e., dopaminergic models (amphetamine) and glutamatergic models (ketamine, PCP, MK-801). Other pharmacological models like neonatal ventral hippocampal lesion model and gestational MAM model are considered as neurodevelopmental models.

#### Dopaminergic model

As dysregulation of dopamine which causes the hyperactivation of the mesolimbic system, is the underlying theory of dopamine hypothesis, animal models are also developed to mimic this feature. For this purpose, dopamine agonists (amphetamine) are used:

##### Amphetamine model

Amphetamine is a psychostimulant, increases the levels of dopamine in synapse. Amphetamine inhibits the dopamine reuptake by acting on dopamine transporter thereby levels of dopamine are increased in synaptic cleft. Dopamine levels are also increased due to the action of amphetamine on vesicular monoamine transporter 2 [35]. Animal model studies revealed that there is involvement of nigrostriatal pathway than mesolimbic pathway. Nigrostriatal pathway is responsible for dyskinetic effects. Mesolimbic system is associated with the locomotor activating effects when amphetamine is given at low doses. Repeated amphetamine administration triggers structural and neurochemical changes which include increased levels of dopamine release from dorsal striatum and the NAcc and the number of dendritic branches and the density of the spine in the NAcc shell and PFC [34]. Impairment in sensory gating observed in schizophrenic patients can also be seen in animal models by assessing the PPI. Amphetamine can impair PPI in rodents (rats). Chronic or repeated administration of amphetamine increases PPI and stereotyped behaviors over time with increased frequencies, which is called sensitization. This sensitization can be seen in both pre-clinical models and humans. Sensitized rats show unimpaired deficits and cognition impairment in pre frontal cortex, extra-dimensional shift and attentional set shifting task [36]. Impairment of LI by amphetamine can be seen in rats and humans. At higher doses, which causes stereotyped behaviors, including sniffing, chewing, and locomotion in rodents as shown in Fig. 1B. When amphetamine administered in monkeys, increased stereotyped behaviors that mimic the paranoid schizophrenia in humans. Also, social isolation can be related as social withdrawal that occurs in humans. This type of sensitization can be prevented by pre-administration of haloperidol and chlorpromazine [37].

### Brain changes associated with amphetamine induced sensitized state

Repeated exposure to amphetamine and its sensitization causes changes in multiple aspects of brain physiology at neurochemical level. Morphological changes such as dendritic branching increases mostly in prefrontal area and decreases in orbitofrontal cortex [38]. Due to this sensitization, the excitability of neurons in the PFC is altered. Sensitization causes decrease in dendritic branching in non-human primates but the concept behind these differences is unclear. Though repetitive amphetamine treatment induces behavioural changes but the mechanism is not clear. Altered PFC function is observed in amphetamine exposed rats that show attentional set shifting. Amphetamine sensitization can also impair the release of acetylcholine in mesolimbic pathway. Due to dysregulation in cholinergic function attentional deficits has been observed [39, 40]. The model has limitation like construct validity is limited between PPI and psychosis. Predictive validity is poor for cognitive and negative symptoms [39].

#### Glutamatergic models

Dysregulation of Glu and NMDA receptors is the underlying theory of glutamate hypothesis which is considered as pathogenesis of schizophrenia. Animal models also mimic this feature by using NMDA noncompetitive antagonists such as ketamine, phencyclidine (PCP) and dizocilpine (MK-801).

##### Ketamine model

Ketamine is a PCP derivative which acts as the uncompetitive NMDAR antagonist and has been developed as the safer alternative of PCP. The administration of ketamine has shown enhancement in the power of gamma oscillations, functional connectivity and prefrontal glutamate (or glutamine) levels which is supported by both animal microdialysis studies and human proton NMR spectroscopy studies. The mechanism behind these manipulations due to ketamine administration is not fully understood but it’s been suggested that NMDAR inhibition causing decline in GABAergic interneuron function, most probably via the preferential effects of ketamine on NMDARs expressed on these cells [36]. Recent findings suggest contrasting results and further makes an assumption that decline in GABAergic interneuron function might be associated with generation of brain superoxide, because decreasing superoxide levels prevented reductions in interneuron activity following ketamine administration, which was also supported by animal model results [41]. Administering acute doses of ketamine instigates schizophrenia like symptoms including positive and negative symptoms and other cognition impairments in healthy volunteers. The downstreaming effects of ketamine such as increased prefrontal glutamate levels and ketamine induced alterations in functional magnetic resonance imaging signals were attributed to positive symptoms, while as ketamine binding to NMDAR has been related with negative symptoms. The acute ketamine may only induce condition closer to the prodrome/early state of schizophrenia. Acute doses have been criticized of not been able to mimic the neurobiological changes that usually is seen in schizophrenia patients and thus, chronic or long term effects of ketamine are being considered which instigates symptoms closer to schizophrenia. Chronic ketamine has been found of causing brain imaging alterations such as reduced thalamic N-acetyl-aspartate (NAA) and prefrontal grey matter volume, and altering the biochemical settings of brain such as upregulation of D1 receptors in frontal cortex as seen in schizophrenia patients [36]. A study using sub-chronic administration of subanesthetic doses of ketamine in brown rats resulted in reduced association of [3H]L-glutamate on glutamate receptors in frontal cortex while as enhanced binding of [3H]L-spiroperidol on D2 receptor in hippocampus. Low doses of ketamine i.e. 10, 20, and 30 mg/kg increased the glutamate release in the PFC, thus triggering the postsynaptic non-NMDA receptors which led to dysregulation of dopaminergic neurotransmission (cognitive impairment) in PFC. The same research revealed the biphasic effect of ketamine on the glutamate flow in PFC i.e. the anesthetic doses (200 mg/ml) suppressing the glutamate levels while as low subanesthetic doses (30 mg/ml) enhancing the glutamate levels in PFC [42]. Employing sub-anesthetic doses of ketamine (5–10 mg/kg) in rodents has also been considered to instigate schizophrenia-like symptoms such as hyperlocomotor activity and cognitive deficits. Recent study investigating the use of acute and chronic ketamine doses administered subcutaneously in rats (Male Sprague Dawley rats) found that acute and chronic ketamine induced variable effects on network connectivity, event-related brain potentials and event-related oscillations, thus association of possible underlying modifications in NMDAR–GABAergic signaling and possible mechanism of how cognitive symptoms are elicited in schizophrenia [33]. Other pharmacological intervention paradigms involves acute ketamine (20 mg/kg intraperitoneal (i.p)) administered in rats inducing decreased memory performance; subchronic ketamine (20 mg/kg, i.p) administered in mice for 7 or 14 days inducing hyperlocomotor activity, decreased sociability and decreased spatial and recognition memory performance; subchronic ketamine (25 mg/kg i.p) administered in rats for 7 days inducing hyperlocomotor activity, decreased no. of social contacts and PPI deficits; and subchronic ketamine (20 mg/kg i.p) administered in mice for 14 days inducing PPI deficits, decreased memory performance and social preference [43].

##### Phencyclidine (PCP) model

PCP induces delusions and hallucination in normal individuals whereas in schizophrenia it causes positive and negative symptoms. In rodents both acute and chronic administration of PCP causes cognition impairment. Chronic PCP causes several neurochemical changes. The micro-dialysis data, shows that chronic administration of PCP decreased DA levels in PFC [34]. Chronic PCP causes pacing behavior in primates whereas in rodents it causes deficits in working memory, stereotyped behaviors, disruption of PPI and changes in mesolimbic dopamine system [36]. In mice and rates, chronic PCP reduces Glu release in PFC. Reduced basal glucose utilization in PFC is seen in chronic intermittent PCP model. This intermittent model reduces the levels of NAA and N-acetyl-aspartyl-glutamate (NAAG) in temporal cortex and enhances the levels of NAAG in HC which in turn causes neuronal dysfunction which is seen in postmortem studies of schizophrenia. Neonatal PCP causes alterations in Glu function by increasing the levels of NMDA subunits like NR1, NR2A, NR2B in frontal cortex [34]. In rodents, sub chronic PCP fails to identify the difference in sugar intake which is used to assess the change in reward and this can be relatable to anhedonia which is seen in schizophrenia. Acute PCP causes behavioral changes which includes hyperlocomotion, social withdrawal and impairment in cognition and PPI. Chronic PCP causes deficits in temporal and frontal lobe which mimics the symptoms of schizophrenia. In rat, chronic PCP (3–21 days) reduces social interaction which can be reversed by administration of haloperidol and clozapine injection. In rodents, irrespective of strain, chronic PCP produces cognition impairment. In rats, pharmacokinetic of PCP are altered due to gender but, sub chronic PCP impairs learning and recognition equally in both genders [44]. The pharmacological intervention paradigms involve acute PCP (10 mg/kg i.p.) or subchronic PCP (10 mg/kg subcutaneous (s.c)) for 10 days administered in mice inducing hyperlocomotor activity, increased immobility time in FST and decreased memory performance while as subchronic PCP (2 mg/kg i.p. 2 × day) administered in rats for 7 days inducing decreased performance in attentional set-shifting tasks [43].

##### Dizocilpine model

Dizocilpine also called as MK-801, which is NMDA non-competitive antagonist. Sub chronic (> 10 mg/kg) administration in rodents causes neurodegeneration and cell death. Various studies show that single dose (4 mg/kg) also can induce the changes in rats [45]. Chronic administration of MK-801 causes decrease in interneuron density in HC [46]. Various studies have proven that MK-801 show 4 to 10 times stronger behaviour in female rats as compared to male rats. It causes molecular changes, modifies the expression of NR1 and NR2 (subunits of NMDA). Also decreases GABAergic parvalbumin (PV) positive interneurons which mimics the brains of patients of schizophrenia [45]. Chronic administration also increases Glu in HC [44] while as Acute administration of MK-801 in rodents causes hyperlocomotion and cognitive impairments [46]. The pharmacological intervention paradigms involve acute MK-801 (0.1 mg/kg i.p.) administered in rats inducing decreased spatial and recognition memory performance; subchronic MK-801 (0.1 mg/kg i.p) for 7 days administered in mice inducing decreased locomotor activity, decreased sociability and PPI deficits; and subchronic MK-801 (1 mg/kg i.p) administered in mice for 14 days inducing hyperlocomotor activity, decreased sociability and PPI deficits [43].

#### Neonatal ventral hippocampal lesion model (NVHL)

Barbara Lipska and colleagues developed NVHL model. A lesion is made at HC region of brain and ibotenic acid is infused into the pups. The pups which are used for this should be in between postnatal day (PND) 6 and 8. Infusion of ibotenic acid causes temporary inactivation on PND 7 which causes schizophrenia like symptoms, including reduced PPI and LI, hypersensitivity to psychostimulants, social interaction deficits, and impairment in cognition during adolescence [47]. NVHL causes functional deficits which are prominent in PFC. Lesioned adult rats also show sensitivity to dopaminergic and glutamatergic stimuli which increases dopamine release in mesolimbic system. Due to these alterations NVHL can cause modifications in signalling pathways in brain regions. Research studies have shown increased locomotor activity of lesion rats may be due to the increased GSK-3 activity. NVHL shows reduced levels of NAA in PFC and GAD67 mRNA expression. Besides, it causes decrease in spine density and dendritic length of pyramidal neurons in both PFC and NAcc [34]. Levels of nitric oxide (NO) in ventral hippocampal region and thalamus, and levels of zinc and metallothionein (MT) in ventral HC are increased at pre pubertal age of NVHL. Whereas at post pubertal age, NO levels are increased in PFC and decreased MT levels in thalamus and NAcc. Increased levels of NO in PFC can cause changes in synaptic communication. Zinc interacts with the glutamatergic receptors and participates in synaptic plasticity [48]. Research studies have shown that NVHL rats tends to consume more alcohol than the control group during adolescence. This neuro developmental model shows high prevalence of alcohol use disorder among patients with schizophrenia [49]. Neurochemical changes and pathways associated with NVHL are depicted in Fig. 1C.

#### Gestational MAM model

Methylazoxmethanol acetate (MAM) is a toxin that inhibits the mitosis in an organism's prenatal brain. Social and cognitive deficits at adulthood are induced when MAM is administered at gestational day (GD) 17. When MAM is given at PND 10, elevated levels of 2-arachidonoylglycerol (2-AG) in brain due to increased expression of diacylglycerol-lipase (DAGL-β) which is a biosynthetic enzyme. Increased 2-AG levels can suppress glutamate release by activating cannabinoid CB1 receptor presynaptically. This also reduces glutamatergic neurotransmission, which causes hypofunction of NMDA receptor and mGlu5 receptor expression, which is also seen in schizophrenia patients [50]. Downregulation of SYP gene and decreased synaptophysin and GAD1 expression in HC and PFC due to the administration of MAM in rats and as well as in schizophrenia patients. When MAM is introduced on GD 17, it causes mitosis inhibition and selectively targets the brain structures, including HC, corpus callosum, and frontal regions. As it targets frontal regions it causes cognitive dysfunctions [51]. MAM treated rats show Stereotyped behaviours in response to the amphetamine [52].

### Non-pharmacological models

#### Pre-natal stress (PRS) model

This model is useful for studying epigenetic mechanisms which are involved in pathophysiology. When adult offspring of mice exposed to stress during pregnancy exhibits schizophrenia like behaviour characterised by hyperactivity, deficits in PPI and social interaction, sensorimotor gating deficits, coping behaviour, spatial learning impairments and stereotype behaviours [53]. PRS mice are characterised by increased levels of DNMT1 and TET1 expression. Researchers suggest that epigenetic modifications such as changes in DNA methyltransferases and TET can cause changes in brain development and maturation. Increased DNMT 1 and 3a in PFC GABAergic interneurons of schizophrenia patients leads to downregulation of GAD67 and brain-derived neurotrophic factor (BDNF) useful for neurogenesis and synaptic plasticity. PRS mice also show decreased expression of mGlu2 and mGlu3 receptors in PFC. Increase in the levels of 5MC and 5HMC which are promoters of schizophrenia related genes are seen in PRS [54]. Increase in levels of 5MC and 5HMC at GAD1 can cause down regulation of BDNF gene expression. Studies have shown that stress during gestation elevates glucocorticoids which inhibits neuronal plasticity by reducing HC long-term potentiation as shown in the Fig. 1D. These levels cause changes in offspring related to biochemical physiological and behavioural. This also induces cognitive deficits in offspring. Dendritic length spine density decreases when male rats are exposed to stress. Researchers have found that changes in proteins such as LIM & SH 3 protein1, prohibit in transferring the hippocampi of prenatal stress rats through microarray techniques. Overexpression of DPYSL2 neurons causes increase in levels of Glu and dysfunction of DPYSL2 causes neurodevelopmental abnormalities [55]. Studies have shown that increased levels of Rab GDIα expression in the HC, when mice are injected with MK 801. Mineralocorticoid receptor and glucocorticoid receptor mRNA levels are downregulated in PRS mice [56].

#### Post-weaning social isolation model (maternal deprivation)

To understand the etiopathology and develop novel drugs with enhanced therapeutic effect, scientists usually develop reliable and predictive animal models of psychiatric diseases like schizophrenia [34]. Thus, Post-weaning social isolation (PWSI) model was designed which could induce symptoms similar to psychotic diseases e.g., Cognitive impairment and other schizophrenia related deficits such as anxiety disorders, apathy, social withdrawal, aggressive behavior etc. Close similarity of neural and behavioral development of rodents with the developmental stages in humans allows rodents to be the best animal model. Different stages of development in rodents e.g. Adolescence is considered in between the period of weaning and early adulthood, while as other developmental stages can also be identified based on behavioral and neurobiological studies [57]. For the sake of study, different stages of development of rodents are identified as pre-adolescence (21st to 28th PND), early adolescence (PND28-PND34), mid adolescence (PND34-PND46), and late adolescence (PND36-PND56). At 56 PND, the stage can be considered as early adulthood. Researchers are using the different stages of rodent developmental phases to study PWSI e.g. Isolation rearing, which is a chronic PWSI where a rodent experiences adverse early life and social deprivation [57].

**Fig. 2:**
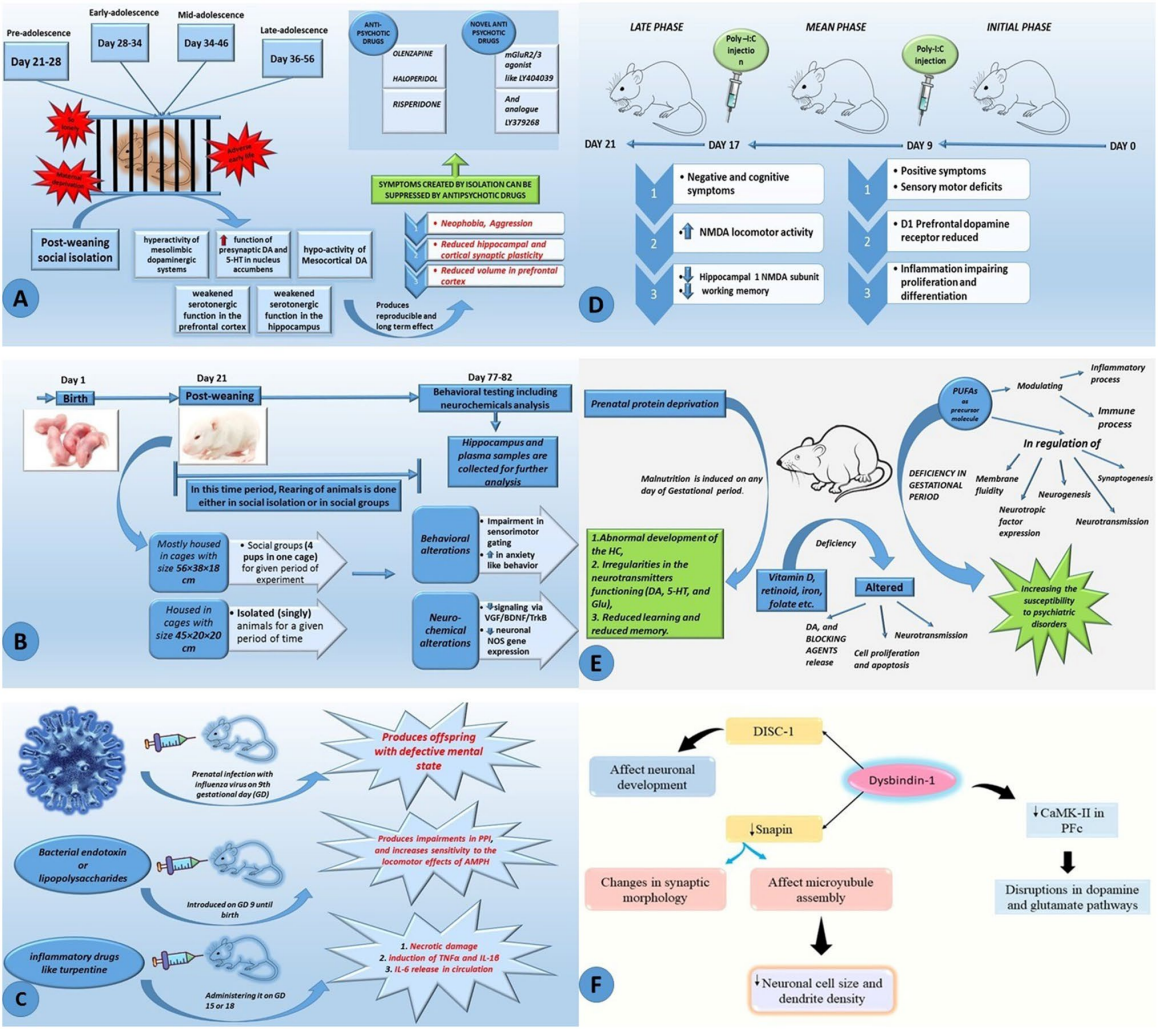
**A** Depicts the causes and effects produced by PWSI and further how to suppress it **B** Depicts the experimental setup and steps involved in isolation rearing mode **C** Depicts different approaches to induce prenatal immune challenges **D** Depicts prenatal exposure of polyriboinosinic-polyribocytidylic acid (Poly-I:C) on 9th and 17th gestational day generates positive and negative/cognitive symptoms respectively of the disorder **E** Depicts the different nutritional deficiency leading to abnormalities in neurotransmitters and neurotransmission **F** represents interaction of dysbindin with different proteins

The collected data from PWSI models of rodents shows hyperactivity of mesolimbic dopaminergic systems, increased function of presynaptic DA and 5-HT in NAcc, hypo-activity of Mesocortical DA, and weakened serotonergic function in the PFC and HC [58]. Maternal deprivation has also caused morphological and biochemical changes in rodents, including the number of neurons in prefrontal and retrosplenial cortex and neural expression in neocortex and HC. Thus, the combined effect of these adverse conditions produces reproducible and long-term effects, e.g. Fear of new things (neophobia), aggression, reduced hippocampal and cortical synaptic plasticity, and reduced volume in the PFC. As these structures play important role in cognitive behavior, it could help the researchers to design the pathogenic pathways of schizophrenia [59]. Comparing these effects with the symptoms of schizophrenia, much resemblance has been seen with the basic signs of the disease. The whole process of post weaning social isolation model, their effects on neural activities and how to reverse the same is briefly shown in Fig. 2A.

As the isolation of rodents (specifically rat pups) causes positive symptoms like hyperactivity within 2–3 weeks, few researchers took advantage to examine the sensitivity of this symptom to antipsychotic drugs.[60]. Other group of researchers have shown that dopamine D3 but not D2 antagonism reduces isolation-induced hyperactivity [61]. The symptoms created by isolation can also be suppressed by giving usual antipsychotic drugs like olanzapine, haloperidol, risperidone and novel anti-schizophrenic drugs, mGluR2/3 agonist, LY404039, and its analogue, LY379268 [58]. Looking at the results of post weaning isolation model causing hyperactivity (due to mesolimbic dopamine hyperactivity) and reversal of the same by using antipsychotic drugs, strongly inclines to its usefulness in studying and validating antipsychotic drugs for schizophrenia.

#### Isolation rearing (IR) model

To produce neurochemical, structural and behavioral manipulations in the brain without any drug administration (non-pharmacological approach), isolation models came into play where rodents are isolated before subjects turn to adulthood. The symptoms induced by IR mostly mimic the clinical symptoms of psychotic diseases like schizophrenia, depression and anxiety [62]. The IR model has the potential of producing dopamine hyperactivity in mesolimbic dopamine pathway resulting in enhanced behavioral sensitivity to dopamine agonists and firing activity of dopamine neurons, elevated dopamine concentrations and altered dopamine turnover in the frontal cortex, elevated basal and amphetamine-stimulated dopamine release in the NAcc and dorsal striatum, and reduced responsivity to dopamine antagonists. The abnormalities caused by IR in HC, thalamus, and frontal cortex of animal subjects includes increased density of 5-HT_1A_ receptors in the HC, reduced synaptophysin immunoreactivity in the dentate gyrus, reduced BDNF in the HC and decreased spine density, depletion of PV-positive GABA interneurons, metabolic abnormalities in the HC and thalamus [63]. These aberrations cause behavioral abnormalities which includes disturbed PPI and sensorimotor gating affecting the cognition of the subject which has been recognized as the basic symptom in patients with schizophrenia. Thus, IR model induced pathological changes help to test novel antipsychotic therapeutics prior to clinical trials [64]. The experimental setup and steps involved in IR model are shown in Fig. 2(B).

Other studies on IR of rodents suggests involvement of two pathways including stress induced neurotoxicity and loss of neurostimulation due to isolation rearing, might collaborate at distinct levels to explain the etiology of brain disorders like schizophrenia. Further encompasses hypothetically the contribution of these pathways causing neural circuit damage or inappropriate development and eventually contributing further to social isolation of the subject [63]. Obscurity in knowing the exact mechanism of how the rodents are being affected by isolation but the neural results of the model are recognized across the different areas of the brain e.g. in amygdala, HC, PFC, and NAcc. Few reports suggest that isolation induces mitochondrial and immuno-inflammatory changes related to cortico-striatal dopamine disturbances and eventually leads to behavioral abnormality [65].

#### Prenatal immune challenge (PIC) model

Abnormalities in behavior, histology and gene expression are characteristics of schizophrenia, that can be attained in pregnant rodents by compromising their immune system which produces anomalies in their offspring. Many methods are used to induce such abnormalities like viral exposure (e.g. Human influenza and borna virus), cytokine releasing agents (lipopolysaccharide bacterial endotoxin) and viral mimetic particles (e.g. Polyriboinosinic-polyribocytidylic acid) [66]. The influenza virus infection to pregnant rodent seems to be helpful in the investigation of known epidemiological-related psychoses in the offspring and many researchers are working to know the cellular and molecular mechanism by which the infection is introduced in utero and that leads to schizophrenia as shown in Fig. 2C [67].

The imbalance in maternal or fetal cytokine levels causing immune activation and dysregulation in mesocortical and mesolimbic dopamine pathways led to the cytokine release models, which links the maternal infection and the development of postnatal behavioral cerebral pathology. To attain the desired models, the rats and mice are exposed to different immunologic stimuli e.g., Viruses, lipopolysaccharides and polyriboinosinic-polyribocytidylic acid as shown in Fig. 2D. In general, PIC models provide substantial evidence for assuming that infection or inflammation caused by prenatal exposure causes neuronal abnormalities leading to psychosis related behavior in humans [67].

Polyriboinosinic-polyribocytidylic acid (poly (I:C)) injection acts through toll-like receptors 3 (TLR3) stimulating activation of many pro-inflammatory cytokines, specifically IFN- α and IFN-β types. An animal studies reported that prenatal administration of poly (I:C) shows increased DA function and impaired cognition in the offspring, constitutes animal model of schizophrenia. [68] Several other changes after administration of poly (I:C) injection includes sensorimotor gating deficits, functional and morphological changes in HC and entorhinal cortex in adult offspring, hype in tumor necrosis factor-α (TNF-α) protein levels in maternal plasma and placenta etc. has been recorded [66]. The outcome of poly (I:C) injection on brain development in pregnant rodents depends upon the dosage and at what stage (gestational period) it is being administered. Generally, the gestational period of rodents is 21 days (on average) and 9th and 17th day of GD is considered important to induce changes such as substantial deficits in PPI, decreased dopaminergic D1 receptors in PFC of the offspring, impaired WM and enhanced locomotor activity were seen mainly in animals when admitting them to PIC [69]**.**

#### Maternal malnutrition model

Maternal malnutrition has been associated with different neuropsychiatric anomalies in infancy, childhood, adolescence, or adulthood. Evidences on maternal malnutrition eventually developing into psychiatric anomalies in adulthood has been seen in Dutch Hunger Winter (1944–1945). Deficiency of many nutrients has been considered the potential candidates linked with schizophrenia, including proteins, fatty acids, vitamins and other micronutrients like retinoid, iron, folate, etc. [70]. Many deficits in the offspring may be instigated by prenatal protein deprivation (PPD) such as abnormal development of the HC, irregularities in the neurotransmitters functioning (DA, 5-HT, and Glu), reduced learning and memory [71]. A hypothetical belief includes that PPD affects many domains of adult’s brain function and development. It would contribute to PPI deficits, one of the core deficits seen in schizophrenia [72]. Few reports support the role of polyunsaturated fatty acids (PUFAs) especially n-3 PUFAs, to increase susceptibility to psychiatric disorders like schizophrenia. The belief is mostly based on requirement of PUFAs as precursor molecules contributing in modulating inflammatory and immune processes, in regulation of membrane fluidity, synaptogenesis, neurotropic factor expression, neurotransmission and neurogenesis [73]. In response to vitamin D deficiency in animal models, altered release of DA and blocking agents, neurotransmission, altered cell proliferation and apoptosis were seen, linked with schizophrenia [74]. All maternal malnutrition approaches are briefly shown in Fig. 2(E). More research is going on animals to enlighten the impact of nutrient deprivation on the behavioral and biochemical properties.

#### Genetic models

##### Dopamine transporter knockout (DAT-KO) mice

Mice lacking Dopamine transporter (DAT) fails to reuptake the dopamine which causes an increase in levels of neurotransmitters in extracellular fluids. Unavailability of DAT in mice elicits variable physiological changes such as the amount of time needed in re-uptaking of DA from the synapse is increased 300-folds, that leads to five-fold enhancement of the extracellular levels of DA. Increased synaptic DA levels results in decreasing the number of DA receptors like D1 and D2 (not D3). Thus, the imbalance of DA in DAT-KO mice can be related to dopamine hypothesis of schizophrenia as these mutants are hyperactive, stereotypic, and show substantial dysregulations in PPI and spatial cognition function [75]. Morphological abnormalities such as changes in dendritic spine of pyramidal neurons and decrease in the density of dendritic spines in medial PFC region of brain are observed in post-mortem studies of schizophrenia patients. The reason behind the changes may be due to dopamine transmission [76]. Psychostimulants such as amphetamine, directly interact with DAT, increases dopamine. Locomotor activity of DAT-KO is associated with TAAR1, which regulates the dopamine transmission and reduces the behavioural effects caused by psychostimulants. Dopamine hyperactivation causes genetic manipulation of DAT, which leads to PPI deficits. This also decreases Y-maze spontaneous alterations i.e., WM. BDNF has been found dysregulated in PFC of DAT-KO mice as DAT deletion reduces BDNF mRNA levels in PFC [77]. This modification induces positive symptoms of schizophrenia. Post-mortem studies have shown the abnormalities in neurons of dendritic spines [78].

##### Catechol-O-methyltransferase (COMT)

COMT inactivates catecholamines and enhances degradation of dopamine in PFC. Meta-analysis has shown that COMT gene which is responsible for schizophrenia like symptoms, is located at the chromosome 22q11.2 [79]. The enzymatic activity of COMT is modified by a guanine (G) to adenine (A) single nucleotide polymorphism known as Val158Met or rs4680, where COMT-Met variant causes significantly decreased enzyme activity instigating increased levels of dopamine in the PFC [80] while as COMT-Val variant has been linked with higher enzyme activity that results in hypodopaminergic states in the PFC and to cognitive disturbances [81]. Both cases end up in dopamine imbalance in PFC and also disturbances in WM and cognition. Other modifications include disturbing dopaminergic signalling include modulation of various dopamine receptors, e.g. striatal overexpression of D2 receptors [81]. COMT is one of the most researched genes in case of schizophrenia and its relation with cognition has been studied. The schizophrenia samples have reported positive and negative associations. Studies show that polymorphism of COMT causes significant decrease in PPI, cognitive defects and delayed memory [82].

##### NR1 and NR2A knockout mice

NR1 knockdown or hypomorph mice show similar behaviors as observed in MK-801 treated animals [75]. Hypomorphic mice with point mutation in D481 (GRIN1) shows deficits in spatial recognition, increased startle activity and impaired hippocampal long-term potential. Deletion of NR1 subunits in mice is neonatally lethal. NR1 KD mice also exhibit reduced NMDA currents, reduced 2-Deoxy-D-glucose uptake in neocortex, increased dendritic length and disrupts the DISC1 protein expression. NR1 gene decreases social behavior especially in pyramid neurons. Reduces the expression of dopamine D2 receptor and GIRK2 in forebrain. NR1 KD mice show gamma oscillations and stereotypic behavior with decreased sensitivity to MK-801 [46]

On the other hand, the NR2A mutant mice represent another form of dysregulated NMDA receptor function. The neurophysiological changes associated with NR2A-KO mice includes impaired DA and 5-HT metabolism in frontal cortex and striatum, increased [^3^H]DA release from striatal slices, an decreased [^3^H]GABA release [75]. The structural dysregulations include deficits in hippocampal long term potentiation (LTP), downregulated NMDAR excitatory postsynaptic currents and LTP in the CA3–CA1 synapse in hippocampal slices. These modifications in NR2A-KO mice induce behavioral changes such as increased locomotor activity in a new surroundings, disturbances in spatial learning, fear coding, and impaired cognition [46].

#### Other mutants

##### Disrupted in schizophrenia 1 (DISC1) genetic mouse models

DISC1 is basically a gene which codes for DISC1, a protein present in synapse and is expressed in early development and plays important role in development of neurons. DISC1 has been associated with schizophrenia and is recognized as the risk factor for it. Studies shows that mostly male rodents are used for studying the DISC1. Increased monoamine levels has been reported in DISC1 mutants [28]. Seven models has been created with inducible or partial loss of DISC1 function, which includes Q31L, L100P, $\Delta$25 bp, BAC-$\Delta$C, CaMK-$\Delta$C and CaMK-cc [83]. Morphologically, these models develop similarities with brain morphology of schizophrenia subjects such as enlarged ventricles, decreased cortical thickness and brain volume. Diminished immunoreactivity for PV has also been reported in the medial PFC and HC [28]. MRI findings of various models also have some supporting results including decreased brain volume in the Q31L and L100P mutants especially in thalamus, cerebellum, cortex and entorhinal cortex, while as in CaMK-$\Delta$C mutant, augmented lateral ventricles have been reported. Also, decreased cerebral cortex and augmented lateral ventricles has been reported in BAC-$\Delta$C. These anatomical and morphological manipulations elicit behavioral change as seen in schizophrenia such as PPI and LI dysregulation [83]. Current genetic findings have compromised the earlier studies of employing the DISC1 transgenic models, as it fails to reflect most of the symptoms of schizophrenia. Meta-analysis and genome-wide association studies have also not supported the association of DISC gene with schizophrenia and are not recognizing it as a potential gene, that could be associated with schizophrenia [84].

##### DYSBINDIN-1

Dysbindin-1 is a coiled protein having cognitive endophenotype which DTNBP1 (dystrobrevin-binding protein-1) gene encodes and is present on chromosome 6p22.3. Dysbindin exists in three isoforms as 1A, 1B, and 1C, which has been recognized as a risk factor for schizophrenia [85]. Dysbindin 1A is mostly located at post synapse, which is believed to be associated with dendritic spine density and causing disturbances in WM in the HC. Dysbindin 1B is a truncated form of 1A expressed in synaptic vesicles involved in glutamatergic transmission and causes deficits in spatial memory. Dysbindin 1C is present in post synapse [86]. The association of dysbindin-1 with schizophrenia causing primary pathology has been related with downregulation of DTNBP1 expression in dorsolateral PFC and HC formation. DTNBP1 gene has been associated with dysregulated cognitive function in both clinical and non-clinical samples [85]. Allelic variation of DTNBP1 has been linked with schizophrenia causing white matter integrity impairments in healthy adult subjects, decrease in grey matter volume in preteenagers, anomalies in neurite outgrowth and morphology, resulting in cognitive deficits [87]. Interaction of dysbindin with different proteins is shown in Fig. 2F. The transgenic dysbindin-1 KO mice shows memory and learning deficiencies**.** Several processes have been reported linking disbindin-1 with schizophrenia such as cognition, glutamate neurotransmission and dopamine neurotransmission. Dysbindin-1 regulates dopamine neurotransmission by impeding release of dopamine and controlling D2 receptor expression. Other reports associate it with regulation of glutamate release, calcium signalling, synaptic vesicle pool size and also NMDA signalling, probably by modifying the expression of NMDA receptor subunits. Biogenesis of lysosome-related organelles complex 1 (BLOC-1) component aggregation, followed by loss of functioning has also been linked with dysbindin-1 overexpression, which could result in loss of Arp2/3 regulation, might contribute directly to dendritic pathology in schizophrenia [86]. Dysbindin also causes copper deficits, which in turn causes impairment in behavior. Alteration in copper levels causes NMDA mediated cell death in HC neurons and inhibits the GABA receptor in cortex [87].

##### NRG1 and ErbB4 knockout mice

Neuregulins (NRGs) are a group of differentiation and growth factors, which are encoded by four genes i.e. NRG1-4. These factors interact with the ErbB, a family of tyrosine kinase transmembrane receptors, especially with ErbB4 is recognized as the chief mediator of NRG1 tasks in the brain. Like other genes and receptors are considered as the candidate risk genes for schizophrenia, both NRG1 and ErbB4 receptor have also been reported. They play an important role in regulating NMDAR signalling and in neurodevelopment. In both human and in rodents, NRG1 is expressed in HC, PFC, substantia nigra and cerebellum, where it modulates the function and expression of GABA, NMDA and cholinergic receptors [46]. NRG1 has almost 30 isoforms, which are mainly divided into six main types especially type III isoform recognized as the greatly expressed NRG1 isoform type in the brain. A transgenic model of this isoform was developed recently exhibited schizophrenia like behaviors such as impaired sensorimotor gating, sociability, and dysregulated fear associated memory. Thus, it has been linked with the potential of developing schizophrenia as NRG1 expressed proteins are associated in synaptic plasticity, myelination and neuronal migration [88]. NRG1 heterozygous KO mice exhibit hyperlocomotor activity, sensorimotor gating deficits and impairments in spatial learning and performance on delayed alternation task. Morphologically, enlarged lateral ventricles and decreased dendritic spine density on HC pyramidal neurons were also seen. While as ErbB4 KO mice also displays similar morphological/behavioral changes such as hyperlocomotor activity, decreased power of kainate-induced gamma oscillations, decreased numbers of PV interneuron density in the HC, decreased calbindin and GABAergic interneurons in the cortex. Current study seems to assume a link between enhanced NRG1-ErbB signaling and reduced NMDAR function in PFC could be a debatable topic with respect to schizophrenia [46].

## Sex differences in animal models of schizophrenia

Animal and human studies show a significant difference in the sex in all aspects, including WM, brain development, positive, negative, and cognitive symptoms. Studies show that male brain becomes masculinized by estrogen during the beginning of second trimester [89]. There is no sex difference in deficits of HC function [90] and working memory [91]. Studies show that, fear conditioning, set shifting, recognition memory, latent inhibition, PPI, social behavior are male specific deficits. Prenatal exposure to bacterial endotoxin is also more in males than females. Exhibiting hyperactivity is more in males, whereas impairment in spatial memory is more in females. No sex differences in cognitive tasks and locomotor activity has been found [28].

## Summary and future perspective of schizophrenia models

With a very complex etiology featured by wide spectrum of physiological/morphological/behavioral manipulations associated with schizophrenia, it is very difficult and challenging at the same time to reproduce any pharmacological or non-pharmacological model which could reflect all the symptoms of this disorder. All the above mentioned models are employed in preclinical studies for discovering any novel drug molecules or profoundly exploring the mechanism of this disorder. Each model has their own advantages and limitations with specific application towards any particular anti-psychotic drug. Generally, all pharmacological models (excluding neurodevelopmental models) are comparatively very simple, inexpensive and time saving procedures than other types of schizophrenia models as the degree of difficulty in making these model is low. They mimic most of the behavioral phenotypes of schizophrenia witnessed in humans including positive and negative symptoms, and other cognitive anomalies. Thus, they are employed occasionally to elicit acute pharmacological modifications which bear resemblance to acute psychosis rather than a permanent dysregulation. As schizophrenia is a chronic disorder with symptoms beginning at the early age, cannot be completely replicated exactly by acute or sub-chronic drug administration. Hence, pharmacological modifications fail to reflect all of the pathophysiological mechanism of schizophrenia which becomes its limitation [43].

As far as neurodevelopmental models are concerned, they reflect most of the symptoms and offer the most advantages over other animal models of schizophrenia. The reason behind this includes development of molecular and behavioral manipulations detected during the post pubertal period, while as no changes during the prepubertal period. The physiological, anatomical and histological changes are observed concurrently with a wide range of behavioral abnormalities, as observed in human subjects. This could be concluded that the changes observed in these animal models are progressive in nature just like observed in human subjects of schizophrenia and can be utilized to understand the pathogenesis of this disorder and possible preventive measures [43]. The chief advantage of these models over other models is the ability to study neurochemical, electrophysiological, and behavioral investigations independent of any surgical intervention, and the ability to analyse reversal effects by therapeutic agents operating on variable pharmacological mechanisms, thus paves the way to discover novel antipsychotic drugs. The limitation includes high degree of difficulty in making the models, requirement of precisely defined days of drug injection in pups and pregnant females of rats, longer duration of waiting before observing the effects of administered drugs and increased cost of maintenance [34].

Finally, the non-pharmacological models (excluding genetic models) can also induce the substantial effects but they are not as efficient as neurodevelopmental models. They are also considered as inexpensive, and simple but needs precisely defined days to isolate the animals in a separate cage. While as genetic models have been created to develop a mutation within a single gene that is associated with the symptoms of schizophrenia. The role of individual proteins can be studied to know its relation with the development of schizophrenia. All of the genetically engineered models are complex, highly expensive, and long lasting processes. Since, the etiopathology of schizophrenia has been correlated with more than one genetic components, which keeps changing over the course of disease. Also, the environmental conditions can influence the development of schizophrenia, thus limits its utility [43].

Until now, usually the animal models generally include animals like rat and mice, are considered because of the resemblances to human physiology, morphology and behavior. But other models have also been discovered including fish mostly zebra fish. It possesses qualities of modeling fewer positive (hyperlocomotion or stereotypy) and negative (thigmotaxis, and reduced social interaction) symptoms, thus restricts its application when modeling the specific symptoms of schizophrenia. This restriction could be attributed to different physiology of humans and fishes. Hence, the zebrafish model remains as the important tool in pharmacology and toxicology, and in behavior studies but remains less explored. Nevertheless, several tasks such as social preference test, mirror biting test etc. studying the social behavior have been created which employs zebrafish [92]. Recently, induced pluripotent stem cells (iPSC) derived neural tissues were used to demonstrate the underlying molecular mechanisms of schizophrenia by employing the neural cell cultures comprised of cells with the same genetic background. While as pluripotent stem cell (PSC) derived brain organoids offered 3-D cortical like structures which reflected the human brain and neuronal complexity. Thus, using iPSC and PSC derived models are recent approaches for developing the models to study neurodevelopmental disorders like schizophrenia [93].

In conclusion, the utility of all these animal models and other recently explored models focuses on to improve understanding of the neurochemical and structural brain modifications that lead to development of schizophrenia and the development of novel antipsychotic drugs. The appropriate animal model should be selected before the beginning of experiments which primarily depends upon the time taken, availability of budget, possible effect of the intervention e.g. any administered drug. At last, the limitations of each model should be remembered while focusing on the advantages, as no animal model had ever entirely reflected the complexity and severity of human disorders.
