# Correction to: Understanding translational research in schizophrenia: A novel insight into animal models

**DOI:** 10.1007/s11033-023-08352-1

**Published:** 2023-04-06

**Authors:** Jonaid Ahmad Malik, Zahid Yaseen, Thotapalli Lahari, Sakeel Ahmed, Mohd Farooq Shaikh, Sirajudheen Anwar

**Affiliations:** 1 Department of Pharmacology and Toxicology, National Institute of Pharmaceutical Education and Research, Guwahati, India; 2 Department of Biomedical Engineering, Indian Institute of Technology Ropar, Rupnagar, India; 3 Department of Pharmaceutical Biotechnology, Delhi Pharmaceutical Sciences and Research University, Delhi, India; 4 Department of Pharmaceutical Sciences, JNTU University, Anantapur, India; 5 Department of Pharmacology and Toxicology, National Institute of Pharmaceutical Education and Research, Ahmedabad, Gujarat 382355 India; 6 Neuropharmacology Research Strength, Jeffrey Cheah School of Medicine and Health Sciences, Monash University Malaysia, 47500 Bandar Sunway, Selangor Malaysia; 7 School of Dentistry and Medical Sciences, Charles Sturt University, Orange, NSW 2800 Australia; 8 Department of Pharmacology, College of Pharmacy, University of Hail, Hail, 81422 Saudi Arabia

**Correction to: Molecular Biology Reports** 10.1007/s11033-023-08241-7

In the original publication of the article, the affiliation “School of Dentistry and Medical Sciences, Charles Sturt University, Orange 2800, New South Wales, Australia” is missed for the author “Mohd Farooq Shaikh”.

The author “Sirajudheen Anwar” is tagged with incorrect affiliation, the correct affiliation of the author is “Department of Pharmacology, College of Pharmacy, University of Hail, Hail 81422, Saudi Arabia”.

The original article has been updated.

